# Identifying ecological corridors for the Chinese ecological conservation redline

**DOI:** 10.1371/journal.pone.0271076

**Published:** 2022-07-08

**Authors:** Meirong Tian, Xiuli Chen, Jixi Gao, Yuxin Tian

**Affiliations:** 1 State Environmental Protection Key Laboratory of Regional Eco-Process and Function Assessment, Chinese Research Academy of Environmental Sciences, Beijing, China; 2 Baotou Teacher’s College, Biological Science and Technology Institute, Baotou, China; 3 Ministry of Ecology and Environment Center for Satellite Application on Ecology and Environment, Beijing, China; 4 Zhengzhou University, Zhengzhou, China; Guangzhou University, CHINA

## Abstract

Due to the fragmentation of protected areas (PA), it is important to build ecological corridors in order to connect scattered PA and form protection networks for biodiversity conservation. We take the Chinese ecological conservation redline (ECR) as an example to study the construction of ecological corridors. China has defined ECR to improve the system of PA in key ecological functional zones, nature reserves, and areas of sensitive and fragile ecological environment. In this study, 187 core areas of ECR were identified using ArcGIS masking and dissolving technology to build corridors, covering 95% of the total ECR areas. Using the Linkage Mapper tool and the Pinchpoint Mapper, we identified 454 ecological corridors 68,794 km long. The results of the line density analysis showed that there are 9 key regional biological corridors connected to the ECR at the national scale, which must focus on protecting and strengthening ecological construction during the implementation of ecological conservation redline policy. Our study will provide references for developing a regional pattern of ecological security, territorial spatial planning, and will promote the implementation of biodiversity conservation policies.

## Introduction

In recent decades, China’s fragmented landscape, blocked ecological corridors and reduced ecological connectivity caused by large-scale regional development have led to spatial isolation of protected areas and “island” effects, directly affecting climate change adaptability and national ecological security [1–3]. Climate change affects the migration of species., e.g., an increase in temperature of 1°C moves the tolerance limit of terrestrial species to the pole by 125 km, or 150 m in the mountains [4]. Every 10 years, European alpine plants advance to higher altitudes by an average of 1–4 m [5]. Constructing a large-scale ecological corridor can bridge species migration, so it was an important solution for biological adaptation to climate change. Moreover, the proportion of species threatened with extinction due to habitat loss and fragmentation is about 48%, 49% and 64% for mammals, birds and amphibians, respectively [6]. Ecological corridors can provide migration routes for animals, increase genetic exchange of species, improve population viability and protect biodiversity by connecting scattered and fragmented animal habitats [7].

Remote sensing and Geographic Information System (GIS) technology have been used to study the influence of anthropogenic activities on ecological corridors [8–11], and to plan regional ecological conservation networks [12, 13]. Methods and models for constructing an ecological corridor include the least-cost path model [14], the gravity model [15], the minimum cumulative resistance (MCR) model [16], etc. Most studies are based on the shortest-path algorithm and GIS spatial analysis to complete multi-scale animal migration corridors [17, 18]. The least-cost path model is widely used in large-scale land management planning, ecosystem restoration and ecological security, as it can identify different types of ecological corridors and extract their spatial information [19–23].

Work on planning and building large-scale international corridors has been rapid, such as the European Green Belt Initiative, the North American Greenway Network, the Mesoamerican Ecological Corridor, the Southwest Australian Ecological Linkage, the Sino-Russian Northeast Tiger Corridor, and the Eastern Himalayas. Therefore, we aim to study the construction of ecological corridors between protected areas in China. Our main aims are: (1) to identify core areas of China’s ecological conservation redline; (2) explore the connectivity of ecological corridors and find the pinch point; and (3) identify key ecological corridors based on GIS line density analysis. Our results can enhance the ecological functions of protected areas and guide the development of conservation networks.

## Materials and methods

### Study area

**Fig 1:**
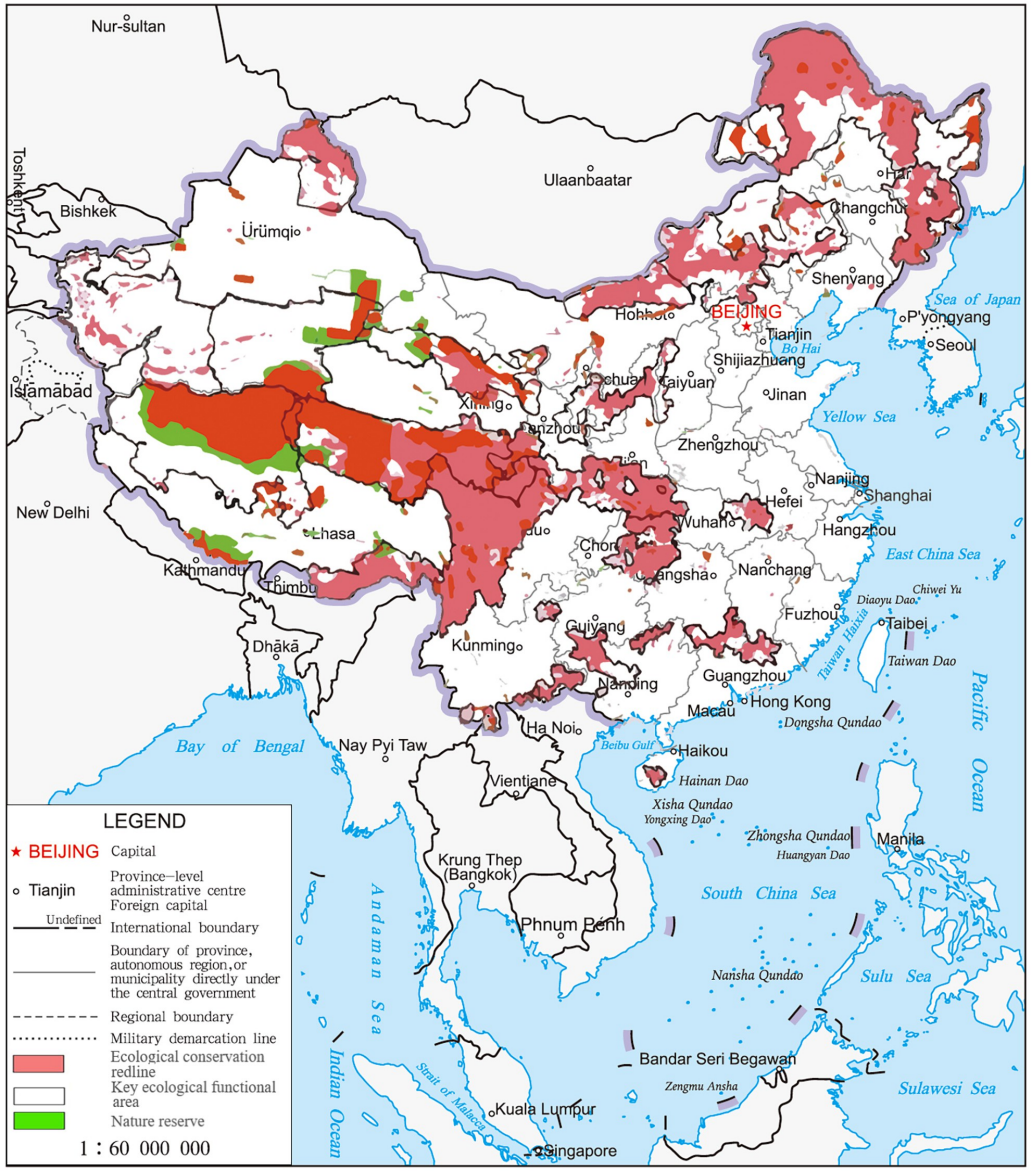
Study area—Ecological conservation redline spatial distribution in China.

China is one of the countries with the richest biodiversity in the world. It has established 11,800 protected areas, covering 18% of the land and 4.6% of the sea area [24]. With economic development, many ecological lands are occupied, which carries potential ecological risks [25]. In 2017, China implemented the “Ecological Conservation Redline” (ECR) program to further improve the system of protected areas (PA), increase their coverage and ensure the welfare of local communities [26–28]. The preliminary designated national ECR area (without marine area) does not make up less than 25% of the land area, covering areas of key ecological functions, ecologically and environmentally sensitive or fragile areas, and key areas for biodiversity protection (Fig 1).

### Methods

**Fig 2:**
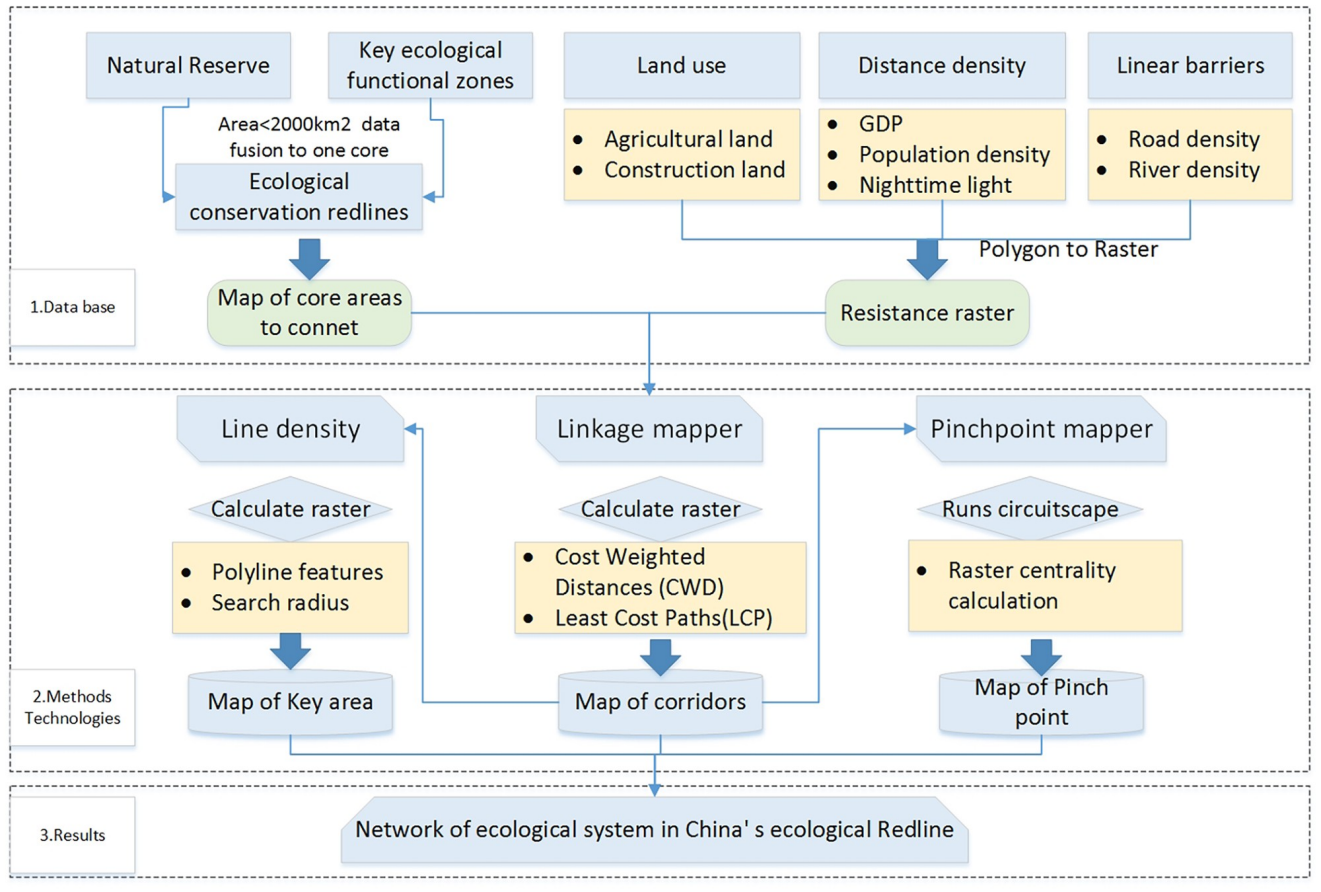
Technical flowchart of the study.

The research method consists of four parts (Fig 2): selection of the core connection area, construction of a comprehensive ecological resistance surface, identification of ecological corridors and identification of pinch point and key areas in the network of ecological corridors.

Land use data were collected from the National Earth System Science Data Centre, while data on vegetation cover, river and road networks were collected from the National Geomatics Center of China. Data on Gross Domestic Productivity (GDP) and population density were obtained from the Chinese county statistical yearbook. Finally, the DMSP/OLS (The Defense Meteorological Program / Operational Line-Scan System) nighttime lights data from 2000 to 2020 were collected from the National Oceanic and Atmospheric Administration, USA.

#### Identification of core areas of the Chinese ecological conservation redline

The ECR have been extracted from key ecological functional zones and nature reserves through spatial masking and dissolving, as well as using data management tool for dissolving and merging. To improve computing efficiency, we reduced the resolution of the ECR to 1 km using the aggregate function in GIS. This has led to a 10-fold reduction in the calculation time of the corridor extraction. The missing data were assigned 9999 and eliminated using the “Eliminate Polygon Part” function of GIS to ensure the feasibility of the ecological corridor calculation. Meanwhile, areas of ≤2000 km^2^ were merged into the core area of the ECR.

#### Construction of the resistance surface

The resistance surface was determined according to the MCR model [16] (Eq. (1)), and the core area, distance and landscape characteristics were comprehensively considered:

$$MCR=f\text{min}\sum_{i=n}^{i=m}(D_{ij}\times R_{i})$$ (1)

where *f* is an unknown positive function reflecting a positive correlation between the minimum resistance distance between any two core areas in space and the landscape characteristics; *D*_*ij*_ is the spatial distance from the core area *j* to the core area *i*; and *R*_*i*_ is the resistance. We have built a three-level system of indicators that includes 11 indicators from three aspects: land use, interference intensity and linear surface resistance. For land use, we considered only Construction land and Agricultural land, while we did not take into account habitat types such as grassland and forest land because they do not have or have less resistance. Indicators are used to construct the resistance surface. Since this study constructs a resistance surface at the national scale, the impact of different species ability to move through the landscape has not been considered. Each indicator was standardized so that its resistance value was between 1 and 100. The resistance surface covering the China’s land was obtained using GIS spatial raster overlay analysis with weights. The weights (w_1_, w_2_, and w_3_) of the resistance factors are weighted using the method of expert scoring based on experience (Table 1).

**Table 1:** Indicator system of resistance factors.

Level-1 indicators	w_1_	Level-2 indicators	w_2_	Level-3 indicators	w_3_
**Land use**	0.4	Construction land	0.6	Urban	0.4
				Rural	0.3
				Other	0.3
		Agricultural land	0.4	Upland field	0.4
				Paddy field	0.3
				Other	0.3
**Disturbance intensity**	0.3	GDP	0.3		
		Population density	0.3		
		Nighttime light	0.4		
**Linear object resistance**	0.3	Road network density	0.5		
		River network density	0.5		

#### Identification of ecological corridors

The ecological corridor is a direct channel for the transfer of materials and energy between the core areas. Ecological corridors were identified using the Linkage Mapper (LM) tool based on the core area and the resistance surface data [29]. The sum of the cost-weighted distance (CWD) raster from each pair of connected core areas was calculated to identify the least-cost distance (LCD) of species migration and diffusion:

$$NLCC_{AB}=CWD_{A}+CWD_{D}-LCD_{AB}$$ (2)

where *NLCC*_*AB*_ is the normalized least cost corridor connecting core areas *A* and *B*, *CWD*_*A*_ is the cost-weighted distance from core area *A*, *CWD*_*B*_ is the cost-weighted distance from core area *B*, and *LCD*_*AB*_ is the cost-weighted distance accumulated by moving along the ideal (least-cost) path connecting a pair of core areas.

#### Identification of Ecological pinchpoint and key areas

Pinchpoint is an area with a high current density in the ecological corridor, which indicates that species are more likely to move through the area between habitats or that there is no alternative path to choose. The pinchpoint is very important and if it is removed or changed, the connectivity can be significantly decreased. The pinchpoint and the key areas in the least cost ecological corridor were identified separately using the Pinchpoint Mapper [30] and line density analysis. The importance of ecological corridors for connecting core areas was evaluated [31, 32] using the Current flow in order to predict the net migration probability of species passing through corresponding nodes or paths, and then to predict areas with high passing level. The current will then flow through these areas between all connected core areas. The results for each core area (all-to-one mode) would be summed in the output current map [32, 33]. Both outputs showed areas that have a high current flow centrality, which indicates their importance for keeping the entire network connected [34]. Therefore, ecological pinchpoint and areas with high line density were considered key areas for the protection of the ecological system and biodiversity.

## Results

### Core areas of ecological conservation redline

**Fig 3:**
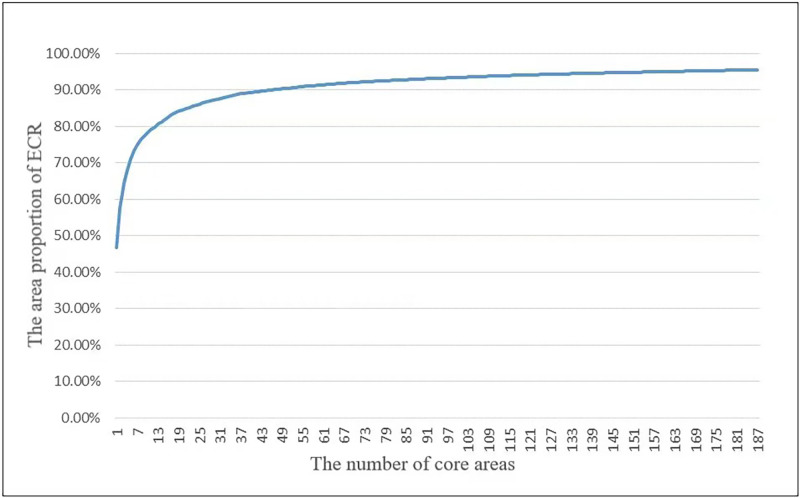
Area accumulation curve selected for the ecological conservation redline.

Spatial analysis methods identified a total of 4446 core areas of the ECR. The number of core ECR areas was then reduced to 2738 (covering 2,575,428 km^2^) using dissolve and merge options in GIS. Based on this, an accumulation curve of the core areas of the ecological conservation redline was created, and 187 core areas of ECR were selected (Fig 3) to ensure that 95% of the ECR area is used for the construction of national ecological corridors.

### Ecological corridors and pinchpoints

**Fig 4:**
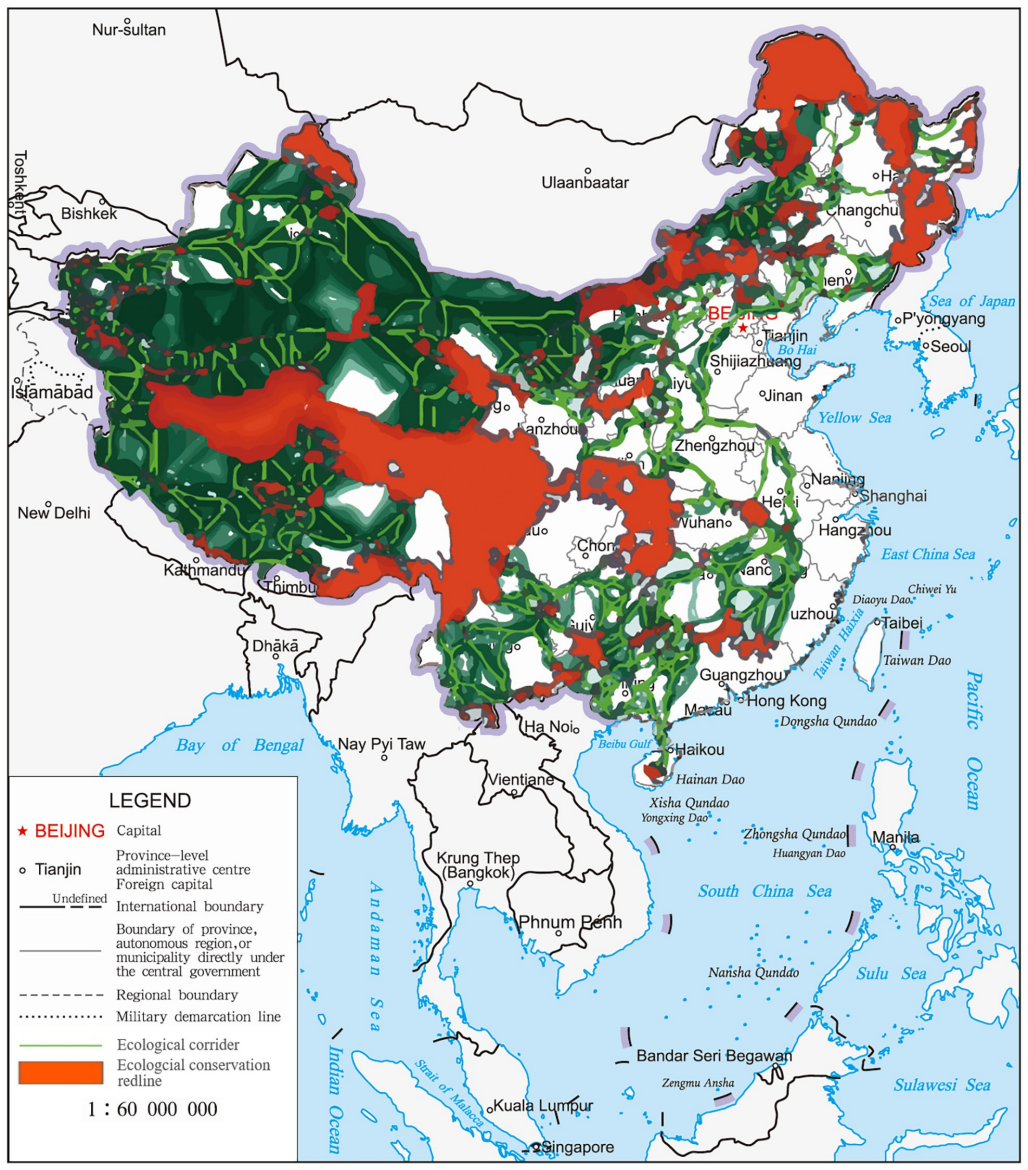
Diagrammatic map of national ecological corridors network based on ECR.

Based on the resistance surface and 187 core areas, we identified ecological corridors using the Linkage Pathways Tool of the Linkage Mapper. There were 454 ecological corridors with a total length of 68794 km (Fig 4). Ecological corridors have connected more than 95% of China’s nature reserves and formed an ecological conservation network. Meanwhile, ecological corridors at the national level were unevenly distributed in space, mainly located in western, southern and northeastern China with good ecological environment and landscape connectivity. Core areas were widespread in Xinjiang, Inner Mongolia, Guizhou, Yunnan, Sichuan, so there were many connections with ecological corridors.

**Fig 5:**
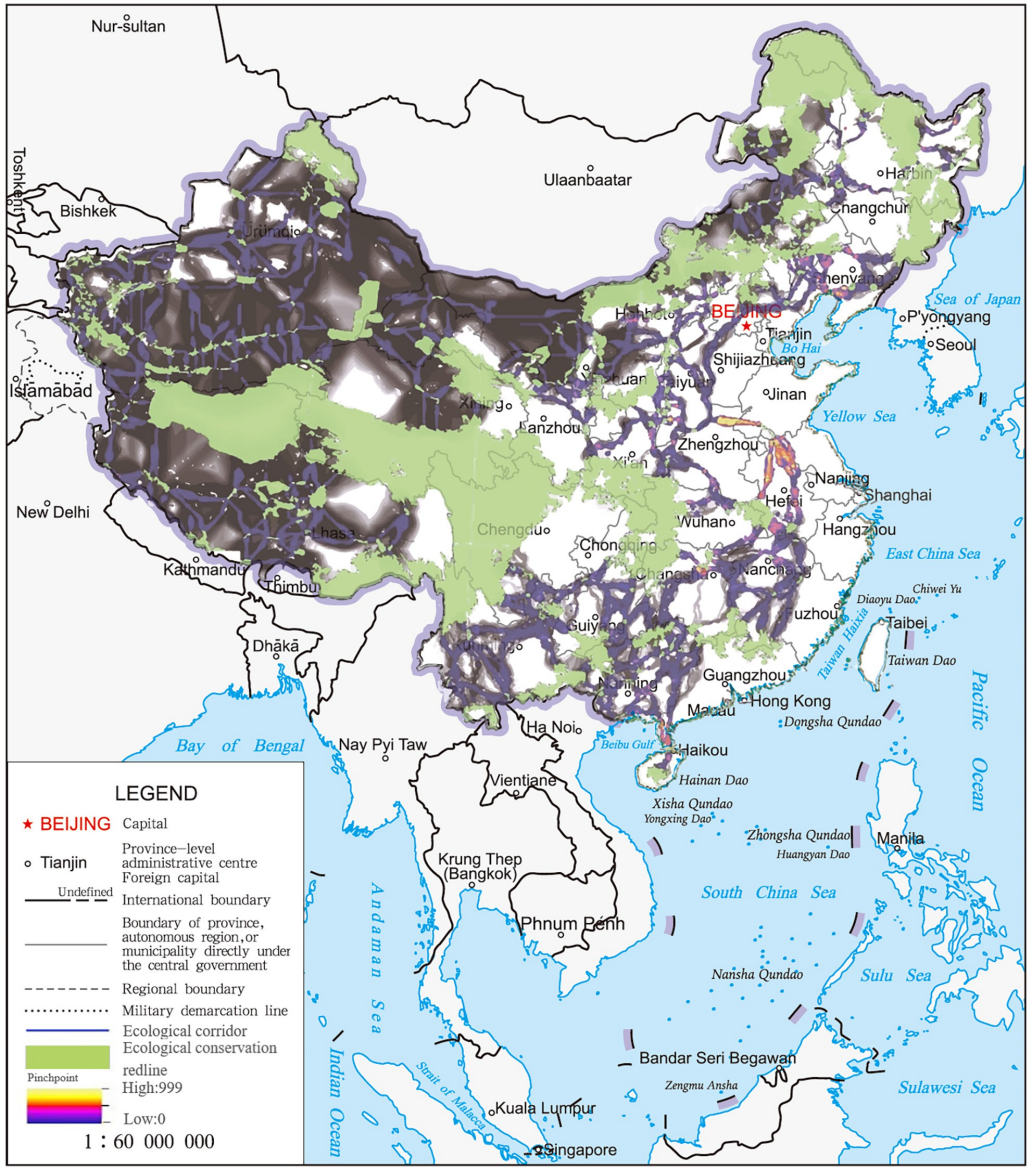
Diagrammatic map of pinchpoint distribution of ecological corridors at the national scale.

There was no overlap of corridors in the network of national ecological corridors, and they maintained national ecological connectivity and ecological functions. Given the overall difference in the contribution of each ecological corridor to national ecological connectivity, we identified a pinchpoint and a key region for maintaining national ecological connectivity. The results showed that almost all ecological corridors between adjacent habitats had a pinchpoint, and some of had narrow strips (Fig 5). Due to the development of the Yangtze River economic belt, the Yangtze River Basin has a large area of pinchpoint distribution, which was considered a "bottleneck" area affecting landscape connectivity and should be dedicated to ecological construction and protection to maintain the connectivity across the PA network.

### Identification of key areas

**Fig 6:**
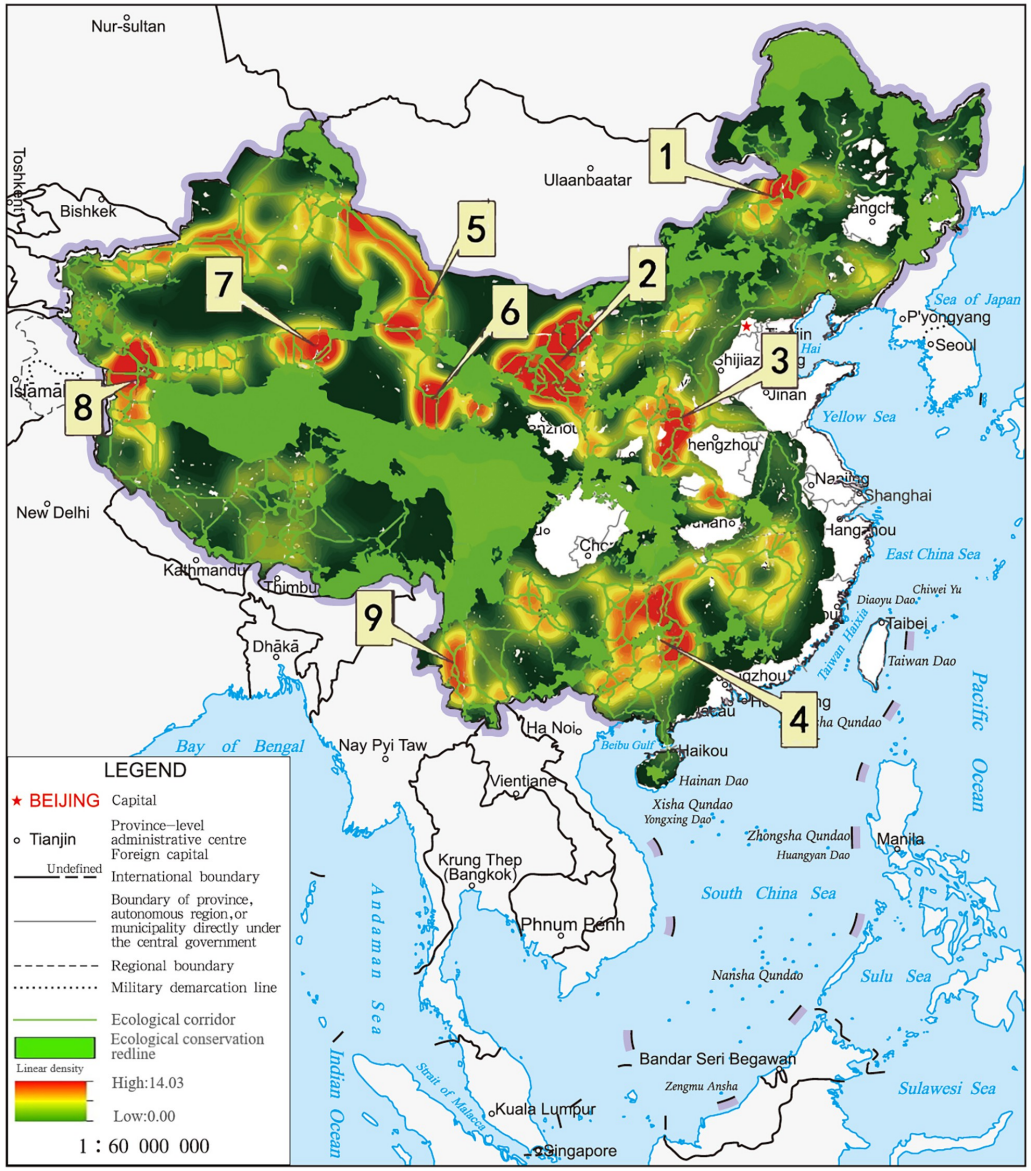
Diagrammatic map of key areas distribution of ecological corridors at the national scale.

The line density analysis shows that there are nine key areas in the Chinese ecological corridor network (Fig 6): (1) the connection area from northern Xilingol to the Hinggan League of Inner Mongolia; (2) the intersection area of the southeastern Alxa League, Jinchang City, Wuwei City, and Zhongwei City; (3) the intersection area of the southeastern Linfen City, the western Luoyang and Jincheng City; (4) the intersection area of Huaihua City, the western Yongzhou, and the northern Hezhou; (5) the intersection area of the eastern Changji and the southeast Hami region; (6) the key area of Jiuquan City; (7) the intersection area of the southeastern Bayingol and the western Qinghai Province; (8) the southwest of the Hotan region; and (9) the longitudinal ridge-valley region in the southwest. During the implementation of the ECR policy, we should focus on the protection and strengthening of ecological construction in key areas.

## Discussion

### Adjusting the core area of ecological conservation redline

The ECR is an original concept and method of environmental protection in China that provides an innovative solution for global biodiversity protection. The study selected the ECR for the construction of ecological corridors and the formation of a network of protected areas that could improve the level of ecological protection and enhance the adaptation of species to climate change [35]. The spatial distribution of ECR in this study is based on the research results and according to the guidelines for delineation of the ecological protection redline [36]. By adjusting the land use structure or the needs of biodiversity protection, the spatial distribution of ECR will be adjusted and ecological corridors will be changed.

### Factors influencing ecological corridor identification

The construction of an ecological corridor generally adopts the least cost path between protected areas with the application of graph and circuit theory [37–39]. The novelty of this study is that we constructed a national network of ecological corridors using LM and obtained a pinchpoint from Pinchpoint Mapper. Consequently, using a line density approach to identify key areas of ecological corridors and maintain large-scale connectivity could contribute to conserving biodiversity facing climate change.

The key factor influencing the corridor distribution is the resistance surface. At present, there is no unified standard for the selection and assignment of resistance factors on resistance surface. In this study, representative indicators such as land use type, river density, road network density, population density, GDP and nighttime light were used as resistance factors to construct the resistance surface. The choice of indicators should be scientific and objective instead of subjective. At present, most landscape resistance assignments are mainly based on expert knowledge or empirical data and there is a lack of experimental data and field surveys on individual dispersal behavior of species [40]. How to reasonably set the resistance value is one of the challenges for the efficient application of the LCP model. Meanwhile, resistance values can be calculated on a grid level using Kriging spatial interpolation technology. This approach can reflect regional differences, but data accuracy was lower due to the lower distribution of national meteorological stations [41].

### Construction of the future ecological corridor

At present, when building an ecological corridor, two aspects need to be considered: i) Will the animals choose ecological corridors for migration? and ii) How to include climate change in the construction of ecological corridors? The MCR model can identify the least cost path between core areas [42, 43], but it cannot reflect the true width of the ecological corridor because it ignores the characteristics of biological random walk [44]. Santos et al. [45] chose ecological corridors with greatest length, width and area and highest forest cover, which were the most important criteria from the ecological functions of corridors. Most studies have not considered the impact of spatial landscape changes on simulation results and the different abilities of species to move through landscape resistance surfaces. The results varied depending on the species and landscape structure characteristics on different spatial scales. The subsequent study will further study the influence of the landscape spatial scale on the simulation of the landscape connection. Furthermore, the analysis of the vegetation types and quality should be considered in the project of ecological corridor construction.

It is more important to incorporate the impacts of future climate change into the construction of ecological corridors, than to react to changes as they occur [46]. To take climate change into account, future standards of functional connectivity need to be studied using simulated movements in hypothetical landscapes due to different climate change scenarios [46]. Meanwhile, field data should be collected to improve connectivity maps [47].

## Conclusion

Eco-environmental problems are gradually showing a trend of regionalization and globalization, and the reduction of the landscape connectivity has become a common problem faced by different regions and countries. There is an urgent need to construct large-scale ecological corridors in order to strengthen the connectivity and integrity of the regional ecosystem and improve the overall service function of the regional ecosystem. This study investigated the construction of ecological corridors based on the core ECR areas and identified 454 ecological corridors at the national level with a total length of 68794 km. The study also identified 9 key areas of ecological corridors for the formation of an ecological conservation network. The location, structure and internal environment of the ecological corridor are key factors in assessing the suitability of the corridor [48]. Therefore, while implementing ecological corridors, government organizations should further assess the suitability of the corridor as it is crucial for the maintenance of corridors functionality [49]. At the same time, in the process of planning and constructing large corridors, it is necessary for the governments of different provinces or countries to provide support for cross-border cooperation.
